# Raw and Purified Clay Minerals for Drug Delivery Applications

**DOI:** 10.1021/acsomega.2c04510

**Published:** 2022-10-18

**Authors:** Gökçe Maide Bekaroğlu, Sevim İşçi

**Affiliations:** Istanbul Technical University, Faculty of Science and Letters, Department of Physics, Maslak 34469, Istanbul, Turkey

## Abstract

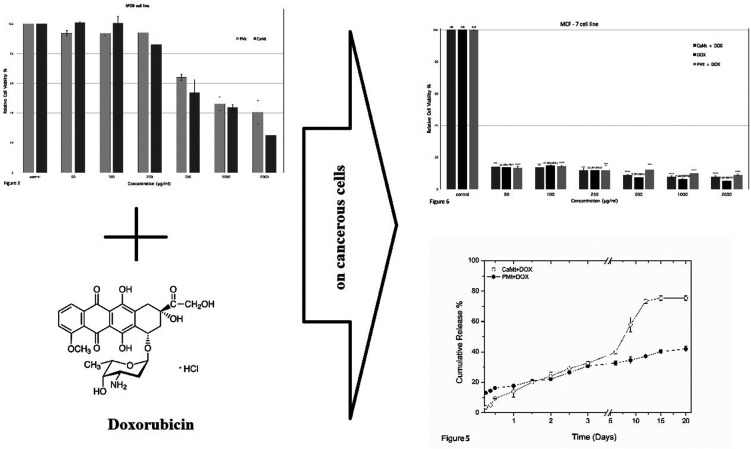

In this study, the aim was to prepare drug-releasing clay mineral particles using raw (CaMt) and purified (PMt) montmorillonite and to compare and determine the effects of purification on the properties of montmorillonite. Montmorillonite clay minerals are used in several pharmaceutical and cosmetic products due to their many favorable properties, such as cation exchange capacity, adsorption capability, high specific surface area, and biocompatibility. Recently, several types of clay minerals have been widely studied for drug delivery applications due to their unique properties. The purification of montmorillonite is considered as a potentially useful step which may decrease the toxicity of impurities but which may increase the adsorption capacity of the montmorillonite. However, the effects on the toxicity and drug-release properties of purified montmorillonite have never been compared to that of raw montmorillonite. Montmorillonite was purified through decomposition of carbonates, dissolution of hydroxides, oxidation of organic materials, dialysis, and sedimentation. The raw and the purified montmorillonite were characterized using XRD, FTIR, and cation exchange capacities. Then, the cytotoxicity of raw and purified montmorillonite on normal hFOB cells was investigated to assess their biocompatibility *in vitro*. Finally, the efficacy of montmorillonite as a drug-delivering agent was investigated *in vitro* using cytotoxicity assays with the MCF7 cell line. The antitumor drug doxorubicin was loaded onto particles through electrostatic forces at 97.99% for CaMt and 96.79% for PMt. The drug-loading efficiency and release behavior of both clay minerals were determined. Results showed that both raw and purified montmorillonite did not significantly reduce the viability of normal cells at low concentrations (<500 μg/mL). At high concentrations, both raw and purified montmorillonite showed significant toxicity and the effect of impurities on toxicity were also more pronounced. Although drug loading was successful for both clay minerals there were differences in their controlled drug-release behavior. Doxorubicin-loaded raw and purified clay minerals significantly reduced MCF7 cell viability similar to pure DOX.

## 1 Introduction

Clay minerals are known biocompatible materials and have been used for medicinal purposes since prehistoric times. Several types of clay minerals have been used extensively to treat pain, open skin wounds, several skin conditions, colitis, diarrhea, hemorrhoids, ulcers, and other gastrointestinal problems.^1^ Recently, montmorillonite clay minerals and their modified forms have also been considered as potential targeted drug delivery agents for administering a therapeutic agent orally to the stomach or colon due to their slow and extended drug-releasing rates.^2−5^ Finally, based on their biocompatibility and drug-releasing rates in such oral applications, montmorillonite clay minerals and their modified forms may also be used for targeted drug delivery to any tumor area by using a special technique called “transcatheter arterial embolization”.^6^

In transcatheter arterial embolization, the flow of the blood supply to the tumor tissue is blocked by microsized (at least 20 μm) particles which are injected into an artery near the tumor or abnormal tissue using a catheter (thin, flexible tube). Consequently, if using montmorillonite, then the success of the treatment would be dependent on the biocompatibility and drug releasing property of montmorillonite. However, information about the potential toxicological effects, biocompatibility, and potential of montmorillonite as an antitumor drug delivery vehicle is rather lacking in the scientific literature.

Moreover, issues regarding the purification and enrichment of clay minerals for medical applications have yet to be addressed adequately in the scientific literature. Currently, it is known that the presence of carbonates or iron oxides in raw clay decreases their biocompatibility as well as their adsorption capacity. Hence, purification would increase biocompatibility and adsorption capacity. In addition to purification, size fractionation is needed to eliminate other clay types from purified montmorillonite and to determine the optimal proportion of montmorillonite.^7^ On the whole, even though purification eliminates imperfections that may be advantageous for eliminating cancer cells (i.e., the toxicity of the imperfections of clay minerals may decrease cancer cell viability), purification results in size reduction which enables increased interaction between the clay surface and the cell membrane and which may result in optimal cell damage.

The comparison and investigation of the effects of raw and purified montmorillonite on cytotoxicity would be significantly useful for future studies on drug delivery applications of montmorillonite. Hence, in this study, the cytotoxicity of raw montmorillonite and its purified form were investigated *in vitro* using normal human fetal osteoblast (hFOB) cells. Also, a cancer drug, doxorubicin (DOX), was loaded onto both raw and purified forms of montmorillonite to compare their drug-loading capacities and release properties. Additionally, the cytotoxicity of drug-loaded raw and purified montmorillonite was investigated on the human breast adenocarcinoma cell line (MCF-7). The results showed that purification reduced imperfections and interlayer spaces of montmorillonite and increased cation exchange capacity. While the adsorption capacity of the clay mineral was increased, however, the reduction of interlayer spaces in purified montmorillonite seemed to restrain the exchange reaction of clay cations with the drug. Fourier-transform infrared spectroscopy (FTIR) results revealed that electrostatic interactions occurred between montmorillonite and DOX. In cytotoxicity studies of raw and purified montmorillonite in normal hFOB cells, purification did not result in any significant effects on the cytotoxicity of montmorillonite at low concentrations. At high concentrations greater than 500 μg/mL, both raw and purified montmorillonite significantly decreased cell viability independently from the composition of the clay mineral particles. In drug-release studies of DOX-loaded montmorillonite, DOX-loaded purified montmorillonite prolonged drug release and showed gradual release in contrast to its raw form. In cytotoxicity studies in cancer cells, DOX-loaded raw and purified montmorillonite treatments showed cytotoxicity almost similar to that of pure drug DOX treatments.

## 2 Experimental Section

### 2.1 Materials

The clay mineral sample was obtained from montmorillonite deposits in Enez, Turkey (Bensan Co.). X-ray diffraction was used to determine the clay mineral types. The dominant clay mineral was found to be dioctahedral montmorillonite with minor amounts of Illite and kaolinite. Quartz was always present in the clay fraction. The montmorillonite was subjected to purification as in Bergaya et al.^7^ The detailed description of the purification process is given below. The raw clay mineral was referred to as CaMt and the purified montmorillonite was labeled as PMt.

Doxorubicin hydrochloride (DOX; (8*s*-*cis*)-10-[(3-amino-2,3,6-trideoxy-alpha-l-lyxo hexopyranosyl)oxy]-7,8,9,10-tetrahydro-6,8,11-trihydroxy-8-(hydroxyacetyl)-1-methoxynaphthacene-5,12-dione hydrochloride) was used as an antitumor drug and was purchased from Sigma-Aldrich.

The purification chemicals Na-citrate dihydrate, sodium bicarbonate NaHCO_3_, sodium dithionite Na_2_S_2_O_4_, hydrogen peroxide H_2_O_2_, hydrochloric acid HCl, and sodium chloride NaCl were purchased from Merck.

### 2.2 Methods

X-ray diffraction was performed using the Bruker D8 Advance model X-ray diffractometer with Ni-filtered and Cu tube XRD at room temperature. The sample was in 2θ ranges from 1 to 15° at a rate of 2°/min.

The chemical composition of the sample was determined by atomic adsorption spectroscopy and silica analysis was performed using the gravimetric method.

FTIR analyses (400–4000 cm^–1^) were performed on the PerkinElmer (Waltham, MA) Spectrum 100 FTIR spectrophotometer using KBr pellets with a sample concentration of 1% (*w*/*w*). Spectral outputs were recorded in absorbance mode as a function of the wavenumber.

Microtrac Nano-Flex particle sizer was used for particle size measurements into the distilled water media.

All the measurements were repeated at least twice to control the results.

### 2.3 Steps of the Purification Process

#### 2.3.1 Dissolution of Hydroxides

Iron (hydr)oxides were removed by complexing the multivalent cations with citrate. First, 1250 mL sodium citrate solution (135 g of Na-citrate dihydrate, 10 g of NaHCO_3_, and 87.5 g of NaCl in 1.25 L of distilled water), 1 L of distilled water, and 300 g of CaMt were mixed with ultra turrax for 1 h. The mixture was then heated to 70 °C, and 60 g of Na_2_S_2_O_4_ was added to dispersion gradually. The color of the mixture changed from brown to blue. The mixture was let to sit for 48 h, and then cooled down and washed twice with distilled water. The mixture was washed with 0.05 M HCl and then twice with 0.5 M NaCl. All procedures were repeated before the next step in the purification process (oxidation of organic materials).^8^

#### 2.3.2 Oxidation of Organic Materials

Small amounts of 10% H_2_O_2_ were added to the wet sediments and heated to 80–90 °C and kept at the same temperature for 2 h. Small volumes of 1 M NaCl were added to the dispersion and then washed with didistilledater.^7^

#### 2.3.3 Sedimentation Procedures

Following the oxidation of organic materials, the wet sediments were dispersed in small amounts into a water-filled sedimentation container. The large-sized particles (greater than 2 μm) settled after 3 days. The supernatants were removed from the sedimentation container and centrifuged.

#### 2.3.4 Dialysis

After the procedures mentioned above, the clay dispersions still contained a considerable amount of salts, mainly NaCl. The excess salt was removed by dialysis. The clay dispersion was placed in dialysis tubes, and the tubes were placed in deionized water which was stirred using a magnetic stirrer. The conductivity of the deionized water was controlled and replaced with fresh water until the conductivity was reached to 6 μS/cm. All sediments were dried using a freeze drier.^7^

### 2.4 Drug Loading to Clay Minerals and *In Vitro* Release Studies

Montmorillonite was loaded with the cancer drug DOX by an adsorption method. DOX (0.8 mg/mL) was dissolved in potassium-buffered saline (PBS, Sigma-Aldrich, St Louis, MO) at pH 5, and the solution was continuously shaken. Then clay particles (2% *w*/*w*) were introduced into the DOX solution, and the dispersions were sonicated for 10 min and shaken with a rotator overnight inside light-protected tubes at room temperature. DOX-loaded particles were referred to as PMt+DOX and CaMt+DOX. After loading DOX to PMt and CaMt, dispersions were centrifuged at 4500 rpm for 10 min, and the supernatants were collected to determine the loading efficiency of the clay minerals. The amount of unloaded DOX in the supernatant was determined by absorption at 480 nm using a UV-spectrophotometer (BIO-RAD Benchmark Plus, Hercules, CA). Drug loading efficiency (LE%) of the particles was determined using the formula below eq 1 (Unsoy et al.):^9^
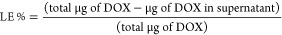
1

*In vitro* drug-release studies of PMt+DOX and CaMt+DOX particles were carried out using PBS at pH 7.4. DOX-loaded clay particles were washed with PBS twice before the beginning of the release studies. PMt+DOX and CaMt+DOX (10 mg) was introduced to 20 mL of PBS separately, and then dispersions were shaken in a rotary shaker at 100 rpm in a 37 °C room for 20 days. At fixed time intervals, sample aliquots of 1 mL were withdrawn and 1 mL of fresh PBS was introduced into the release media. Suspensions were centrifuged to separate particles and released DOX concentrations were obtained from sample aliquots using an UV–vis spectrophotometer (at 480 nm). Cumulative drug-release percentage was calculated from the calibration curve equation of DOX. Each experiment was replicated at least three times.

### 2.5 Mammalian Cell Culture

In this study, both cell lines used were cultured using the same mammalian tissue culture protocol. Cells were cultured in high-glucose Dulbecco’s modified Eagle’s medium (DMEM, Gibco) supplemented with 10% FBS (fetal bovine serum, HyClone), 1% penicillin/streptomycin (Sigma-Aldrich, St Louis, MO), and 1% l-glutamine solution (Sigma-Aldrich). Incubations were performed in a 5% CO_2_ at 37 °C (BINDER C150 E2) humidified incubator. All cells were isolated using trypsin 0.25%/EDTA 0.02% (PAN-Biotech P10–019100) and redispersed in DMEM. All cell cultures were performed with medium renewals, 2–3 times per week.

### 2.6 Cytotoxicity Assays

*In vitro* cytotoxity assays were carried out for PMt, CaMt, PMt+DOX, and CaMt+DOX particles using hFOB and MCF-7 cell lines.^10^ Both cell lines were purchased from American Type Culture Collection (Manassas, VA). Particles were incubated with each cell line at different concentrations to assess their effect on cell viability. Test particles effect on cell viability was determined using Cell Counting Kit-8 (CCK-8, Dojindo Laboratories, Kumamoto, Japan), which contains tetrazolium salt (WST-8, [2-(2-methoxy-4-nitrophenyl)-3-(4-nitrophenyl)-5-(2,4-disulfophenyl)-2*H*-tetrazolium, monosodium salt]). Formazan dye produced by WST-8 was quantified from absorbance at 450 nm using an ELISA multiwell spectrophotometer (BIO-RAD Benchmark Plus, Hercules, CA). The relative cell viability (%) was calculated as follows (eq 2):
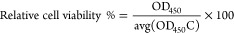
2

Various concentrations of PMt and CaMt particles were incubated with hFOB cells in 96-well plates separately to assess their effect on normal cell viability. Also, MCF-7 cells were treated with various concentrations of PMt+DOX and CaMt+DOX particles to investigate antitumor activity in these cells. Cytotoxicity assays were carried out, after optimizing growth of the cell cultures, using 96-well plates where cells were seeded at 10^4^ cells/well. Control wells were prepared to compare viability using only cells in DMEM. Viable cells were quantified before each assay using a hemocytometer and the trypan blue exclusion method. Cell cultures with various concentrations of test particles were incubated inside a humidified incubator containing 5% CO_2_ at 37 °C for 48 h, and then each well was washed with filtered PBS 2 times to remove the test substances. Then, 100 μL of new culture medium and 10 μL of CCK-8 were added directly to each culture well then incubated for 4 h. Cell viability (%) of all treated cells relative to the control well was calculated from absorbance values at 450 nm using the relative cell viability (%) formula given above. Each treatment was triplicated per plate, and each experiment was replicated at least 3 times.

### 2.7 Statistical Analysis

All statistical analyses were performed using the Statistical Package for the Social Sciences (SPSS) program, and the results were provided with mean ± standard deviation (SD) values. For cell viability assays, statistical differences were obtained using the Student’s *t*-test. Significance values were designated as follows: *, *p* < 0.05; **, *p* < 0.01; ***,*p* < 0.001; and ****, *p* < 0.0001 (vs control).

## 3 Results and Discussion

### 3.1 Properties of Raw and Purified Clay Minerals

The purification process of CaMt changed its chemical composition, interlayer spaces, and surface and adsorption properties. The chemical compositions of both CaMt and PMt are shown in Table 1.

**Table 1:** Chemical Content Analyses (wt %) of CaMt and PMt

sample	SiO_2_	Al_2_O_3_	Fe_2_O_3_	Na_2_O	CaO	K_2_O	MgO	MnO	TiO_2_	P_2_O_5_
CaMt	60.18	18.49	5.65	1.41	4.60	2.31	2.39	0.11	0.68	0.37
PMt	62.3	19.55	4.72	1.89	0.65	0.84	2.04		0.66	

**Figure 1:**
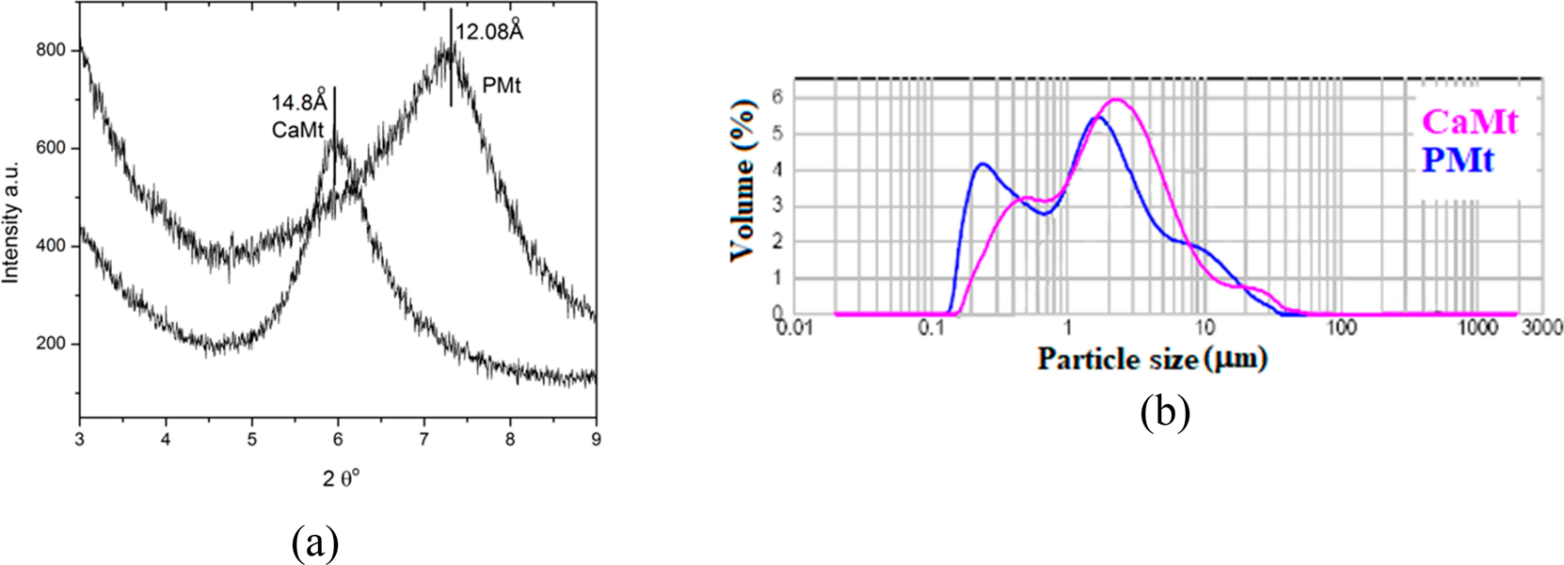
(a) XRD diagram of CaMt and PMt. (b) Particle size distribution of CaMt and PMt.

The interlayer spaces of both CaMt and PMt were determined using XRD analysis. Figure 1 indicates that exchangeable Ca ^+2^ cations of CaMt were replaced by Na^+^ atoms during purification. As a result of this exchange, the interlayer spaces decreased from 14.8 *Å* to 12.08 *Å*. Besides, the average particle sizes of CaMt and PMt were measured as 5.47 and 2.86 μm respectively by dynamic light scattering measurements.

The cation exchange capacity (CEC) of CaMt and PMt was determined to be 7.7 × 10^–4^ and 1.03 × 10^–3^ eq/g respectively using the methylene blue method. These values indicate that the adsorption capacity of CaMt increased after purification. Both CEC and XRD analysis showed that purification of CaMt enhanced the adsorption capacity due to the removal of carbonates, iron oxides, and organic materials form the clay mineral and also reduced the size of CaMt.

### 3.2 Effects of Raw and Purified Clay Minerals at Varying Concentrations on hFOB Viability

**Figure 2:**
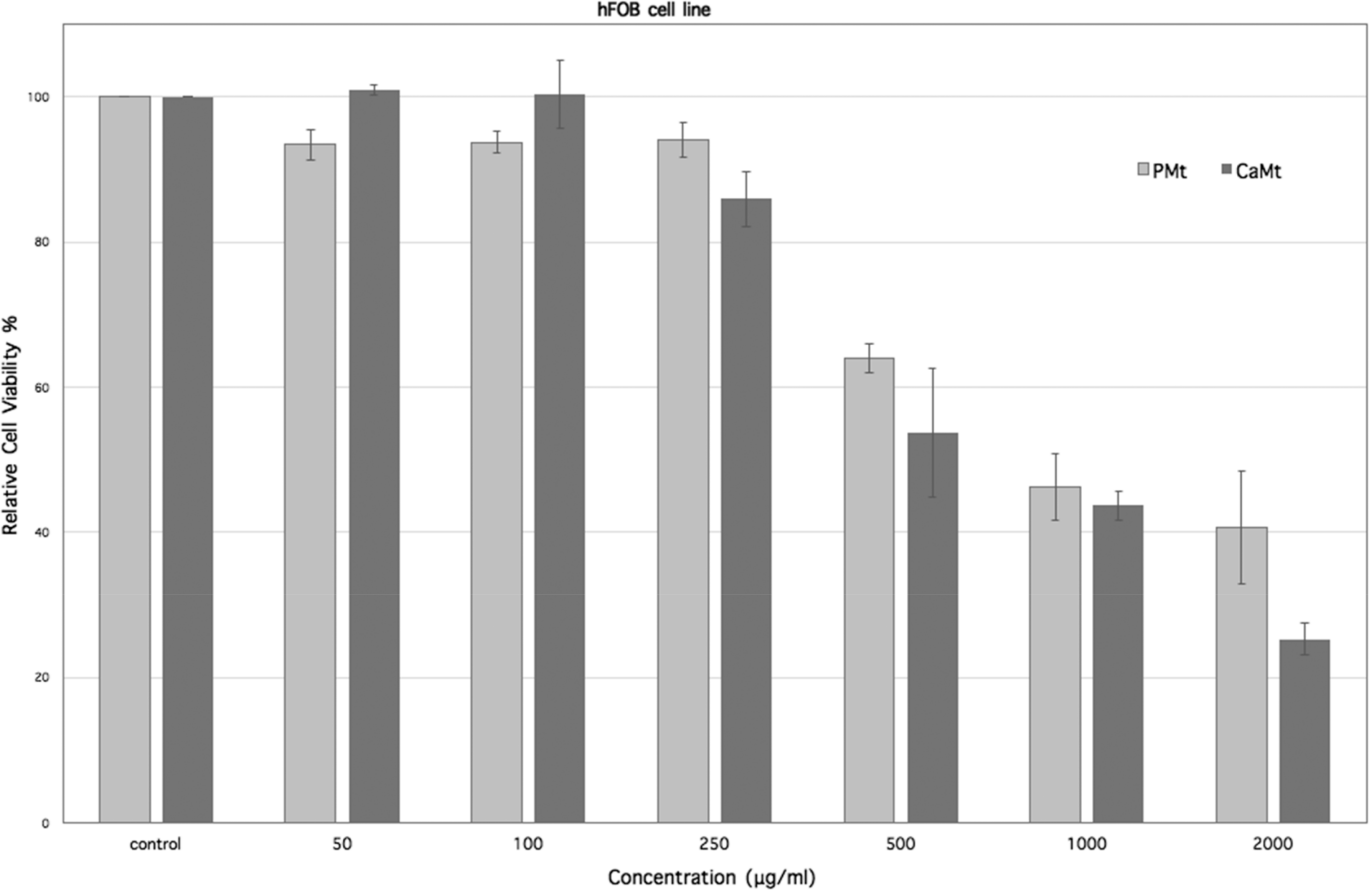
*In vitro* relative cell viability of hFOB treated with CaMt and PMt dilutions (50–2000 μg/mL) (*, *p* ≤ 0.05 vs control).

The effects of both CaMt and PMt on hFOB viability was examined to assess biocompatibility at various concentrations; results are given in Figure 2.

At low concentrations, neither of both clay-mineral-treated cultures showed significant toxicity (*p* > 0.05 vs control). CaMt cultures even showed slightly enhanced cell proliferation at low concentrations. The results revealed that CaMt treatments showed no significant toxicity up to 250 μg/mL concentration, and PMt treatments showed no significant toxicity up to 500 μg/mL to hFOB cell cultures. At these low concentrations, PMt showed higher toxicity compared with CaMT. This difference in cell viability between CaMt and PMt treatments may be due to size and surface area differences rather than the composition of the clay minerals. After purification, the carbonates, iron oxides, and also the organic materials were removed from CaMt, reducing the size of the clay minerals. Size fractionation also further reduced the size of the clay minerals and removed the different type clay minerals from raw CaMt. Thus, the reduced particle size of PMt increased the surface area as well as the interactions between cell membrane and the clay mineral surface, and this might be the reason for increased damage or disruption of membrane integrity of cells from PMt as compared with CaMT treatments at low concentrations.

At high concentrations greater than or equal to 500 μg/mL, both CaMt and PMt treatments showed a significant reduction in cell viability (*, *p* < 0.05, or **, *p* < 0.01 vs control). The decrease in cell viability at high clay mineral concentrations may be due to membrane-damage effects of clay mineral particles which result in necrotic cell death rather than apoptosis. The results are in line with^11^ Geh et al. who reported that at higher tested concentrations, 2/3 of all tested bentonite samples caused lysis of liposomes due to direct interaction between cell and particles independent from the composition of the clay mineral. Also, Geh et al. reported that increased CEC and adsorption abilities may lead to membrane damage and cause cell death due to environmental perturbations. Contrary to the findings at low concentrations, the toxicity of CaMt on hFOB cell cultures was greater than that of PMt at high concentrations, especially at 2000 μg/mL. This difference in toxicity between both cell cultures may be due to the higher number of imperfections in CaMt such as carbonates, iron oxides and other clays in the raw clay mineral which decrease cell viability.

### 3.3 Drug Adsorption and Release Properties of Drug-Loaded Raw and the Purified Clay Minerals

**Figure 3:**
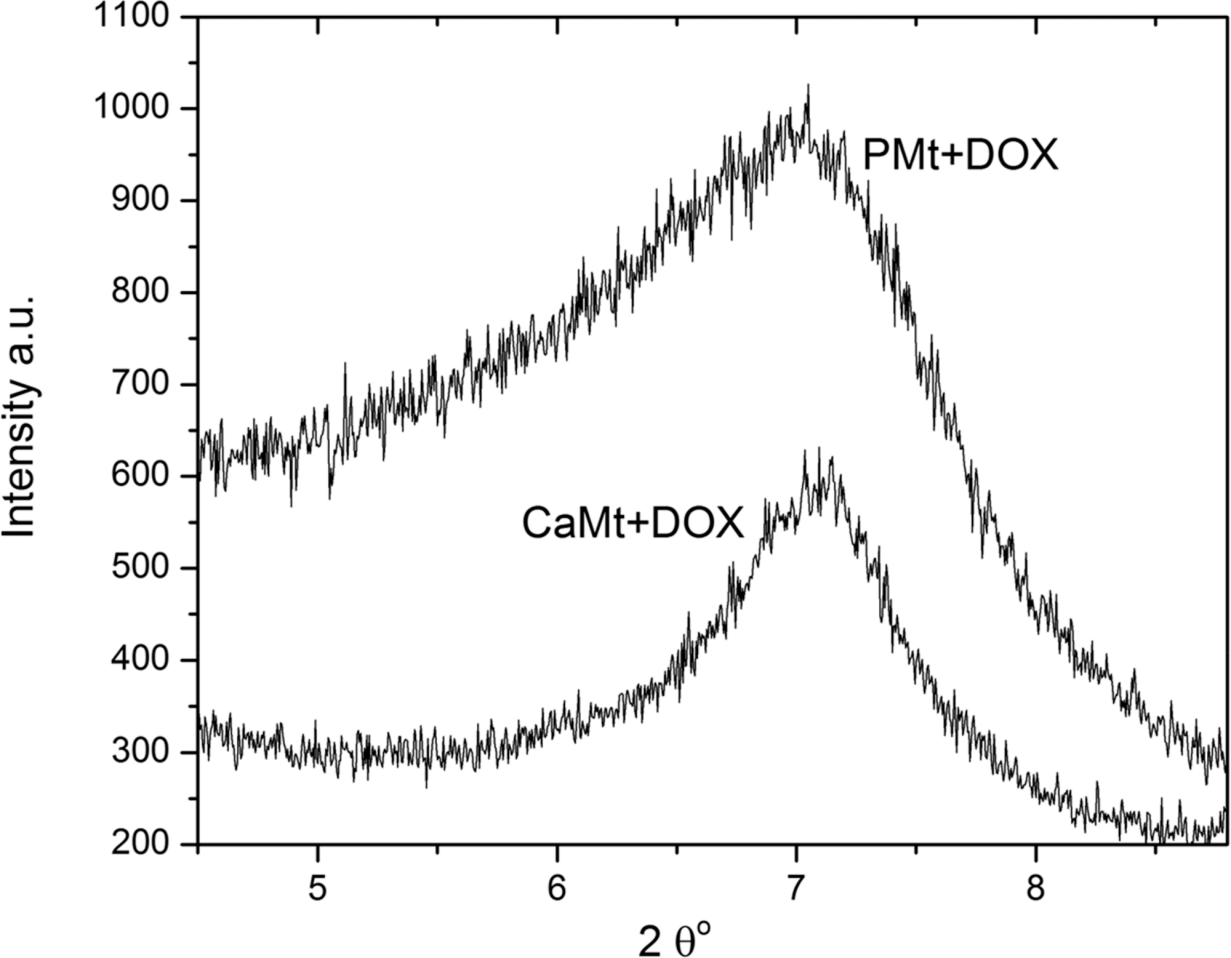
X-ray diffraction diagram of DOX-loaded CaMt and PMt.

DOX is a widely used positively charged cancer therapy drug. Since montmorillonite has negatively charged surfaces, DOX is attracted to the surfaces of clay minerals through electrostatic forces. Moreover, the exchangeable cations of montmorillonite can be replaced with DOX and can increase the interlayer spaces. 0.8 mg/mL DOX was loaded to each clay mineral and adsorption amounts were determined to be 0.784 mg/mL (97.99%) for CaMt and 0.774 mg/mL (96.79%) for PMt. Surprisingly, the drug adsorption of CaMt was determined to be higher than PMt. This could be the result of reduced interlayer spaces of the purified clay mineral. Although CaMt has lower CEC than PMt, CaMt has greater interlayer spaces than PMt, so the drug could easily penetrate in the interlayer spaces and DOX could be attracted more strongly to exchange surfaces, which would result in a more efficient cation exchanging reaction and the adsorption of the drug being increased. The XRD diagrams of drug loaded of both CaMt and PMt were given at Figure 3. The peaks of both sample were found at the same position, and the interlayer spaces of both the raw and the purified samples were determined to be 12.4 Å after DOX loading. The XRD peaks of original samples changed places because of the exchangeable cations for both clay minerals were replaced by DOX, and the interlayer spaces of the clay minerals reached equal distances.

**Figure 4:**
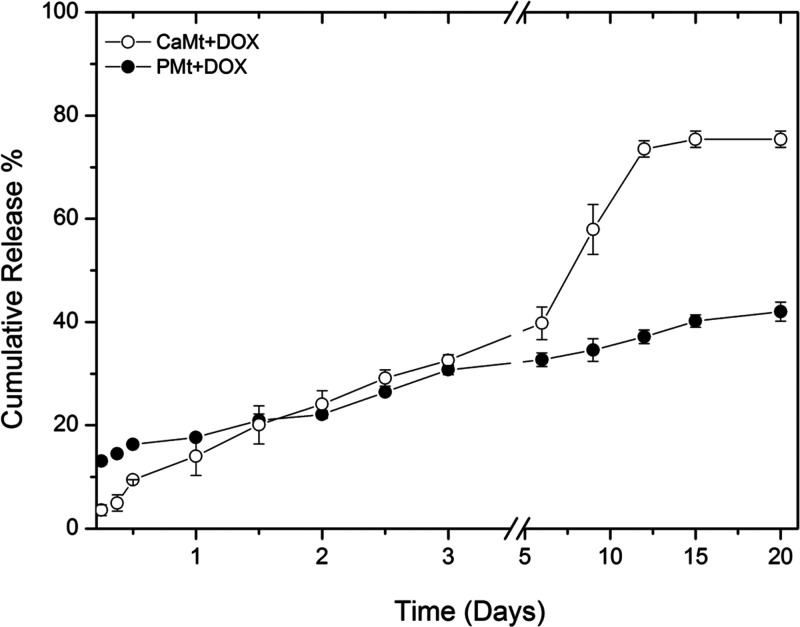
Cumulative drug release (*in vitro*) versus time of DOX-loaded clays, CaMt and PMt at pH 7.

Drug release rates of both PMt+DOX and CaMt+DOX were observed for 20 days at pH 7.4 (physiological pH). The cumulative release in percentage is shown for both clay minerals over 20 days at Figure 4. Initially, PMt+DOX exhibited a burst release behavior (less than 18 ± 1.3% of the loaded drug) followed by gradual release.^12,13^ Incomplete release within 20 days (42 ± 1.8% of the total loaded DOX) was observed for PMt+DOX, which indicates that PMt+DOX particles would still release DOX even after 20 days. Unlike PMt+DOX, CaMt+DOX exhibited a slow initial release of drug followed by a more rapid release after 6 days. The overall cumulative release of CaMt+DOX was 75.4 ± 1.6% of the total loaded DOX amount at day 15 and remained constant thereafter.

The initial burst release behavior of PMt+DOX may be due to its highly specific surface area where its larger surface area allowed more DOX to be at the surface of the clay particles.^2,14^ The increased rate of release of CaMt+DOX after 6 days may be due to external media which led to sufficient dilution for DOX to be released from the interlayer spaces more easily. These results indicate that CaMt+DOX’s interlayer DOX was released more quickly into the media than PMt+DOX, and if allowed sufficient time and dilutions, then PMt+DOX could begin the release of interlayer DOX at a further time.

**Figure 5:**
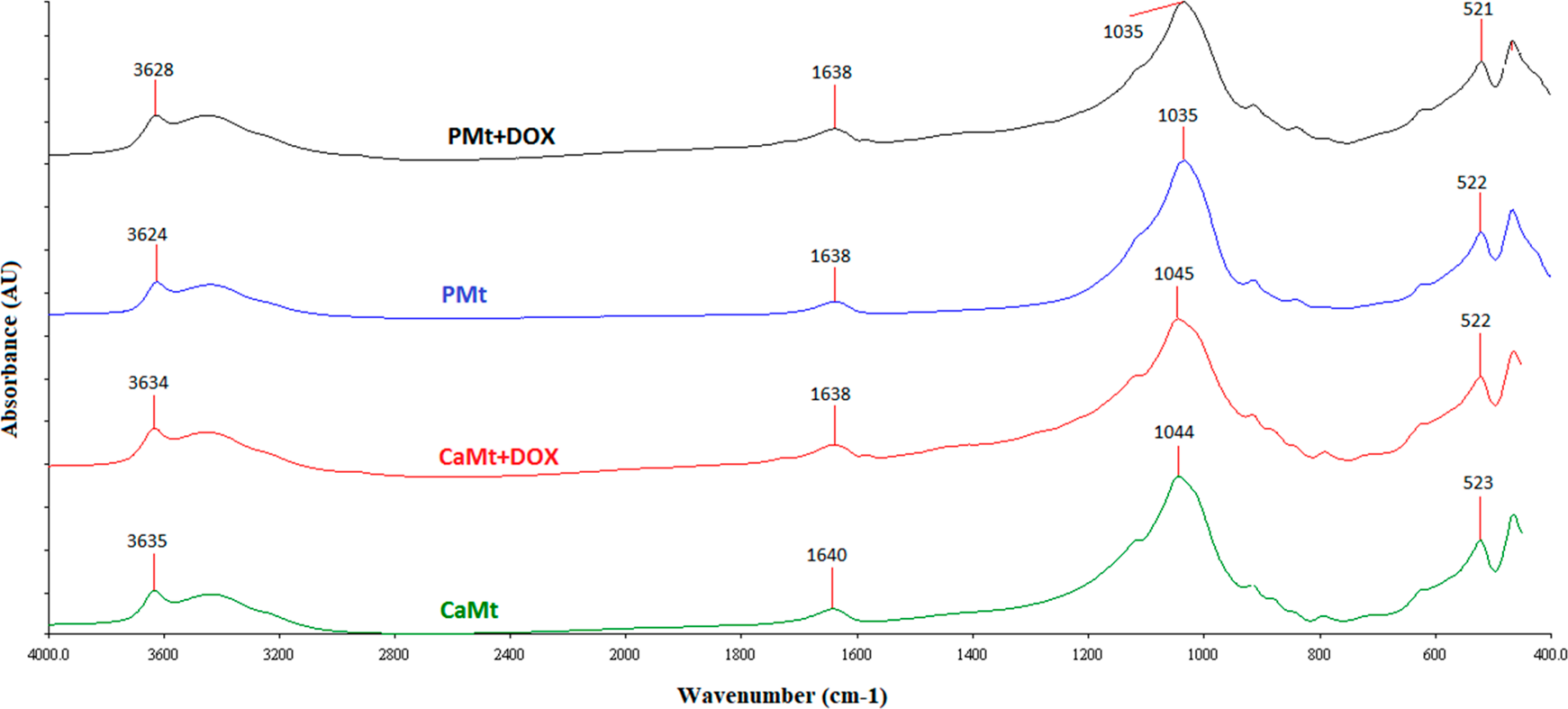
Fourier transform infrared spectra of CaMt, PMt, DOX-loaded CaMt and DOX-loaded PMt.

FTIR spectra of CaMt, CaMt+DOX, PMt, and PMt+DOX, given in Figure 5, were shown to investigate the nature of interaction between the clay minerals and DOX. Characteristic montmorillonite peaks of CaMt and PMt were obtained at 3635 and 3624 cm^–1^ (structural OH groups), 1044 and 1035 cm^–1^ (characteristic Si–O stretching), and 523 and 522 cm^–1^ (characteristic Si–O bending peaks). The spectra of DOX-loaded clay minerals showed insignificant changes at the characteristic peaks which indicates the presence of electrostatic interactions.^15−17^

### 3.4 Effects of Drug-Loaded Raw and Purified Clay Minerals on the Viability of Cancer Cells

*In vitro* cytotoxicity assays were carried out for DOX-loaded clay minerals using the MCF-7 cell line to assess and compare the anticancer drug potential of CaMt+DOX and PMt+DOX particles. The amount of pure DOX was adjusted to be equal to the amount of DOX loaded onto clay mineral+DOX particles.

**Figure 6:**
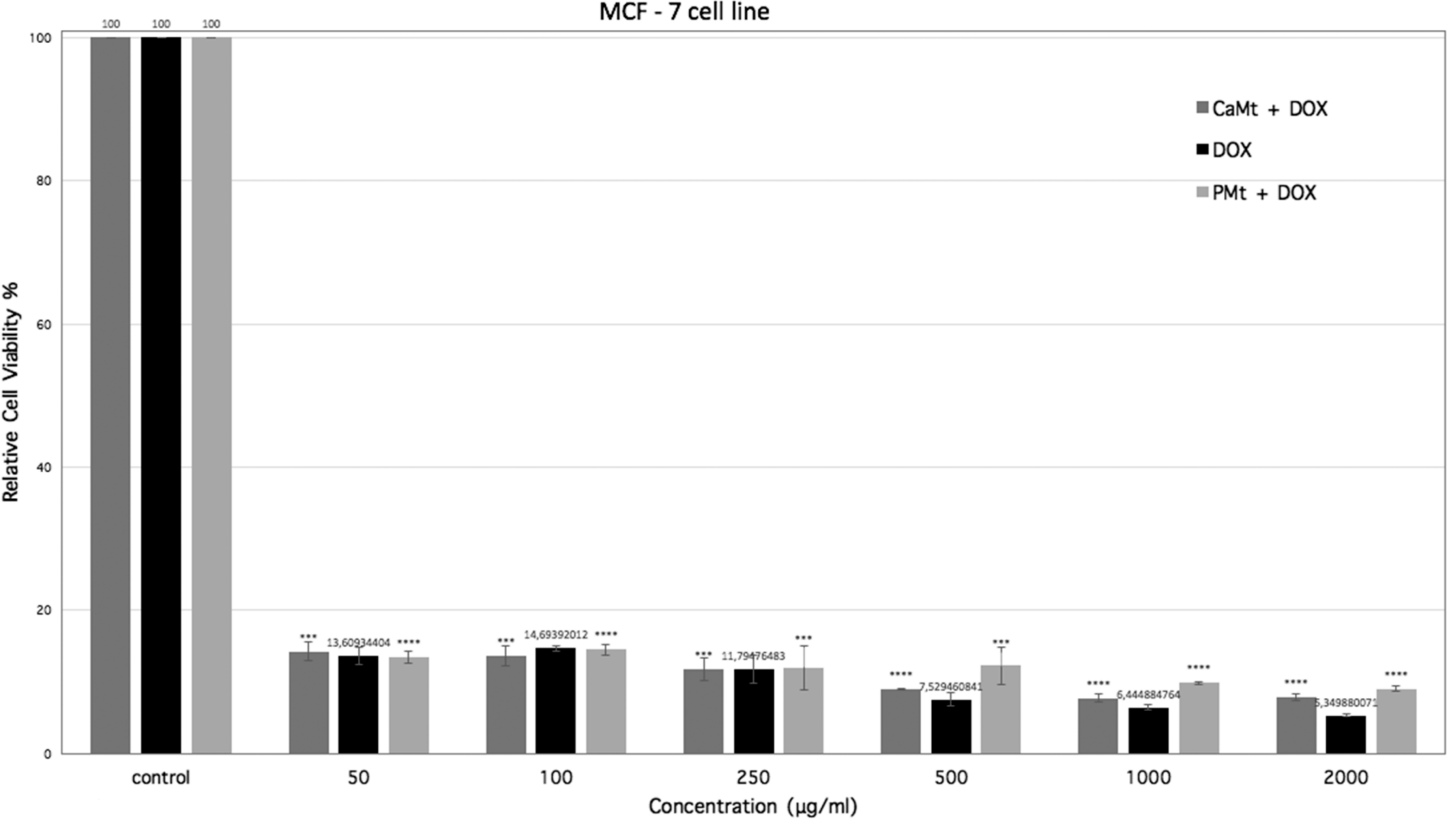
*In vitro* relative cell viability of MFC-7 treated with pure DOX, CaMt+DOX, and PMt+DOX dilutions (50–2000 μg/mL) (***, *p* < 0.001; ****, *p* < 0.0001 vs control).

Relative cell viability (%) of MFC-7 treated with pure DOX, CaMt+DOX and PMt+DOX dilutions were given at Figure 6. Pure DOX and DOX-loaded clay minerals exhibited similar toxicity when administered to MCF-7 cells. DOX and DOX-loaded clay minerals significantly reduced MCF-7 viability at all concentrations (***, *p* < 0.001, or ****, *p* < 0.0001 vs control). As pure DOX amount was adjusted to the loaded amount, and as at all concentrations both DOX-loaded clay minerals exhibited toxicity similar to that of pure DOX, this indicates that DOX-loaded clay minerals were as effective as pure DOX. All concentration treatments resulted in a highly significant reduction to MCF-7 cell viability. At higher concentrations, CaMt+DOX treatments showed slightly lower cell viability than PMt+DOX where this difference could be due to impurities and cell membrane damage effects of the CaMt clay itself, similar to CaMt and PMt results in hFOB incubations.

## 4 Conclusions

Purification of raw montmorillonite resulted in altered chemical composition, interlayer spaces, and surface and adsorption properties. Purified montmorillonite showed reduced impurities, reduced interlayer spaces and increased CEC. However, even though purified montmorillonite exhibited increased CEC, its DOX adsorption was slightly lower than that of raw montmorillonite. This could be the result of reduced interlayer spaces which served to restrain the exchange reaction of clay cations with the drugs. *In vitro* drug-release studies showed that PMt+DOX and CaMt+DOX released DOX differently over 20 days (at pH 7.4). PMt+DOX exhibited a low burst release at initial stages followed by gradual release. The total cumulative release for PMt+DOX reached only 42 ± 1.8% of the total loaded DOX at day 20 which indicated that PMt+DOX would still release DOX thereafter. In contrast to PMt+DOX, CaMt+DOX exhibited a slow initial release of drug followed by a more rapid release after day 6, and the total cumulative release of CaMt+DOX reached 75.4 ± 1.6% of the loaded DOX amount at day 15 and remained constant thereafter.

In cytotoxicity studies in normal cells, hFOB cell viability assays showed no significant toxicity for both raw and purified montmorillonite at low concentrations. Slight differences in cell viability at low concentrations of both raw and purified montmorillonite might be due to CEC, size, and surface area differences rather than the composition of the clay minerals. However, at concentrations greater than or equal to 500 μg/mL, both raw and purified montmorillonite treatments showed a significant reduction in cell viability. Increased toxicity at higher concentrations might be due to increased direct interactions between cell and clay surface, CEC, and adsorption abilities. In addition, the toxicity of impurities in raw montmorillonite treatments was enhanced at high concentrations, and CaMt’s toxicity was greater than that of PMt at these concentrations, especially at 2000 μg/mL. The results suggest that purification is not helpful to reduce toxicity to healthy cells in higher concentration applications of montmorillonite but may be useful in lower concentration applications.

In cytotoxicity studies in cancer cells, DOX-loaded montmorillonite showed similar toxicity to pure DOX treatments whether montmorillonite was purified or not. At higher concentrations, CaMt+DOX treatments showed cell viability slightly lower than that of PMt+DOX due to impurities and cell membrane damage effects of the CaMt clay itself, similar to results in normal cells. For DOX-loaded montmorillonite applications, both raw and purified montmorillonite would be as effective as pure DOX, yet their release behavior should be considered before choosing which is most suitable for the specific application.

Consequently, the results revealed that purification does not significantly increase either the biocompatibility or DOX adsorption amount of montmorillonite clay particles. Purification supplied smooth and prolonged drug releasing property, but the total release was still incomplete after 20 days. Considering the high cost and duration of purification, the results suggest that it is unnecessary for drug delivery applications.
